# Effect of glucose and insulin supplementation on the isolation of primary human hepatocytes

**DOI:** 10.17179/excli2019-1782

**Published:** 2019-11-18

**Authors:** Georg Damm, Gerda Schicht, Andrea Zimmermann, Christiane Rennert, Nicolas Fischer, Melanie Kießig, Tristan Wagner, Victoria Kegel, Daniel Seehofer

**Affiliations:** 1 Department of Hepatobiliary Surgery and Visceral Transplantation, University Hospital, Leipzig University, Leipzig, Germany; 2 Saxonian Incubator for Clinical Translation (SIKT), Leipzig University, Leipzig, Germany; 3 Department of General-, Visceral- and Transplantation Surgery, Charité University Medicine Berlin, 13353 Berlin, Germany

**Keywords:** human liver, primary human hepatocytes, hepatocyte isolation, hepatic energy metabolism, cytochrome P450

## Abstract

Primary human hepatocytes (PHHs) remain the gold standard for *in vitro* investigations of xenobiotic metabolism and hepatotoxicity. However, scarcity of liver tissue and novel developments in liver surgery has limited the availability and quality of tissue samples. In particular, warm ischemia shifts the intracellular metabolism from aerobic to anaerobic conditions, which increases glycogenolysis, glucose depletion and energy deficiency. Therefore, the aim of the present study was to investigate whether supplementation with glucose and insulin during PHH isolation could reconstitute intracellular glycogen storage and beneficially affect viability and functionality. Furthermore, the study elucidated whether the susceptibility of the tissue's energy status correlates with body mass index (BMI). PHHs from 12 donors were isolated from human liver tissue obtained from partial liver resections using a two-step EDTA/collagenase perfusion technique. For a direct comparison of the influence of glucose/insulin supplementation, we modified the setup, enabling the parallel isolation of two pieces of one tissue sample with varying perfusate. Independent of the BMI of the patient, the glycogen content in liver tissue was notably low in the majority of samples. Furthermore, supplementation with glucose and insulin had no beneficial effect on the glycogen concentration of isolated PHHs. However, an indirect improvement of the availability of energy was shown by increased viability, plating efficiency and partial cellular activity after supplementation. The plating efficiency showed a striking inverse correlation with increasing lipid content of PHHs. However, 60 h of cultivation time revealed no significant impact on the maintenance of albumin and urea synthesis or xenobiotic metabolism after supplementation. In conclusion, surgical procedures and tissue handling may decrease hepatic energy resources and lead to cell stress and death. Consequently, PHHs with low energy resources die during the isolation process without supplementation of glucose/insulin or early cell culture, while their survival rates are improved with glucose/insulin supplementation.

## Impact Statement

Primary human hepatocytes (PHHs) are still considered the gold standard for *in vitro* investigations and are valuable resources in the field. However, their usage is limited by the availability of high-quality human liver tissue. Therefore, further adaptation and optimization of liver cell isolation procedures are crucial. In the current work, we showed that the viability and adherence of PHHs could be improved by supplementation with glucose and insulin during the isolation procedure. The data also revealed for the first time that different isolation procedures lead to a selection process on hepatocyte subpopulations displayed by varying hepatic functions. This information is crucial because it shows the impact of the isolation procedure on the functionality of PHH cultures. Consequently, we recommend supplementation with glucose and insulin because it improves the majority of PHH batches and leads to a cell suspension that better reflects the PHH populations of the whole liver lobule.

### Abbreviations

4-MU: 4-methylumbelliferone

BCA: Bicinchoninic acid assay

BSA: Bovine serum albumin

BMI: body mass index

CYP: Cytochrome P450 enzymes

DMSO: Dimethyl sulfoxide

FBS: fetal bovine serum

G6P: glucose-6-phosphate

GK: glucokinase

GLUT2: glucose transporter 2

GST: glutathione-S-transferase

HLCH: high lipid containing hepatocytes

LLCH: low lipid containing hepatocytes

MCB: monochlorobimane

NEAA: non-essential amino acids

PBS: Phosphate-buffered saline

PHHs: primary human hepatocytes

ROS: reactive oxygen species

SRB: sulforhodamine B

w G+I: with 11 mM D-Glucose and 40 IU/l human insulin

WME: William's Medium E

w/o G+I: without 11 mM D-Glucose and 40 IU/l human insulin

XTT: 2.3-Bis-(2-Methoxy-4-Nitro-5-Sulfophenyl)-2H-Tetrazolium-5-Carboxanilide

## Introduction

Primary human hepatocytes (PHHs) are an essential cellular tool in hepatology and toxicology. These cells are used in hepatocyte transplantation, which is under investigation as an alternative therapeutic strategy to orthotopic liver transplantation in selected liver disorders. Additionally, these cells are applied in bioartificial liver devices in acute liver diseases, either as a bridge to transplantation or to maintain liver functions until the liver tissue has regenerated.

PHHs are also useful as an *in vitro* tool in medical, pharmacological and toxicological research. The cells are still considered the gold standard for the evaluation of *in vitro* metabolism and hepatotoxicity during the development of novel drugs and chemical compounds. Moreover, hepatocytes have been broadly used in physiological and pathophysiological model systems for investigations on hepatic functions in health and disease. A major drawback in the use of PHHs is their limited availability in high quality. The tissue originates from explanted diseased livers (Kleine et al., 2014[20]; Lloyd et al., 2004[25]; Richert et al., 2004[32]), rejected livers not used for liver transplantation (Lloyd et al.; 2004[25]) and liver resection specimens (Kegel et al., 2016[17]). However, surgical procedures with prolonged warm ischemia times due to the Pringle maneuver, laparoscopic liver resection (Horner et al., 2016[15]) or preoperative portal vein embolization (Kluge et al., 2016[21]) may impair PHH quality (Lloyd et al., 2004[25]; Richert et al., 2004[32]) or even limit the source of PHHs. The outcome of the cell isolation procedure itself depends on donor characteristics (e.g., blood values, body mass index (BMI), and liver inflammation), tissue processing and cell isolation conditions (Meng et al., 2016[28]; Richert et al., 2004[32]). In particular, the time delay between hepatectomy and the beginning of liver perfusion has been discussed as the most critical factor influencing the success of PHH isolation (Bhogal et al., 2011[2]). This time frame is characterized by warm and cold ischemia times *in vivo* and *ex vivo,* and a correlation of increasing ischemia times with decreasing PHH yield and viability has been shown (Lee et al., 2014[24]).

Moreover, PHH quality can be influenced by dedifferentiation associated with the loss of physiological and morphological functions during cultivation. This process is already initiated in the isolation procedure due to the damage of cell contacts and considerable ischemia reperfusion injury (Vinken et al., 2012[37]).

Hepatocytes play a major role in systemic energy metabolism and supply the organism with metabolites during starvation periods (Rui, 2014[33]). When hepatocytes are stimulated with insulin, glucose can be converted to storage lipids and glycogen (Knobeloch et al., 2012[22]). Blood glucose enters the hepatocytes via glucose transporter 2 (GLUT2) and is phosphorylated by glucokinase (GK) to generate glucose-6-phosphate (G6P) in the cells. This leads to a reduction of the intracellular glucose concentration and a further increase in glucose uptake. In the fed state, G6P acts as a precursor for glycogen synthesis. Alternatively, G6P might also be metabolized during glycolysis and further completely oxidized to maintain the intracellular ATP level (Rui, 2014[33]).

Warm ischemia during surgery and the cell isolation procedure shift the intracellular metabolism from aerobic to anaerobic conditions, thereby increasing glycogenolysis and cellular membrane damage (Isaksson et al., 2011[16]). Therefore, prolonged ischemia reduces intracellular glucose levels and depletes glycogen storage, resulting in a fasted state of the liver. Low levels of glycogen will affect the cellular defense against reperfusion injury (Younes and Strubelt, 1988[38]) and cell regeneration during the attachment phase in cell culture. In hepatocyte culture, an insulin-supplemented medium has been shown to improve PHH attachment and morphology (Fraczek et al., 2013[9]). Therefore, the aim of the present study was to investigate whether the supplementation of the perfusate with glucose and insulin during PHH isolation could reconstitute intracellular glycogen storage and has a beneficial effect on the viability and functionality of PHHs. Furthermore, it was elucidated whether the susceptibility of the energy status and the glycogen level of the tissue and cells correlate with the BMI.

## Material and Methods

### Culture media and chemicals

The hepatocyte attachment and culture medium was based on William's Medium E with GlutaMAX™ (WME, Gibco, Paisley, UK), supplemented with 10 % fetal bovine serum (FBS, Merck Biochrom, Berlin, Germany), 15 mM HEPES, 0.1 mM non-essential amino acids (MEM NEAA 100x), 1 mM sodium pyruvate, 100 U/100 µM penicillin/streptomycin (all provided by Gibco, Paisley, UK), 80 IU/l human insulin (Lilly Deutschland GmbH, Bad Homburg, Germany) and 1 µg/ml dexamethasone (Fortecortin^®^, Merck, Darmstadt, Germany). Phosphate-buffered saline solution supplemented with calcium and magnesium (PBS) was purchased from Gibco and Trypan Blue was provided by Biochrom (Berlin, Germany). All other chemicals were purchased from Sigma-Aldrich (Munich, Germany), if not stated otherwise. Rat tail collagen was prepared in our laboratory according to the protocol established by Rajan et al. (2006[31]).

### Tissue samples

Liver tissue samples were obtained from macroscopically healthy tissue that remained from resected human liver of patients with primary or secondary liver tumors or benign local liver diseases (Table 1(Tab. 1) and Supplementary Table 1). Informed consent of the patients for the use of tissue for research purposes was obtained according to the ethical guidelines of Leipzig University Hospital and Charité University Medicine Berlin. A part of the tissue samples was snap-frozen and stored at -80 °C for later characterization.

### Isolation of hepatocytes

**Figure 1:**
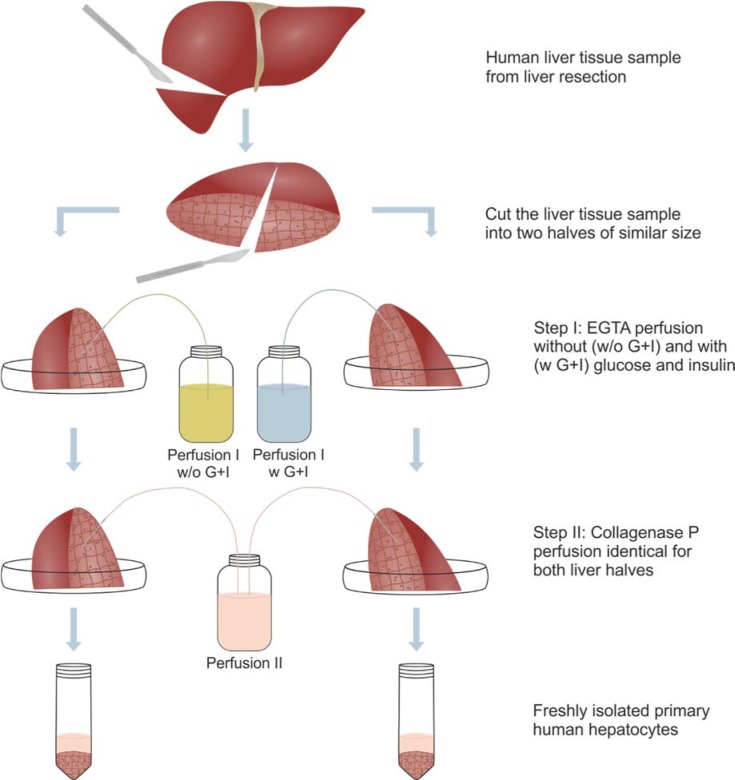
Schematic illustration of the experimental procedure. Every human liver tissue sample was dissected into two almost equal parts. Each liver piece was cannulated to first perfuse it in parallel with an EGTA-containing Perfusion solution I either without (w/o G+I) or with (w G+I) 11 mM D-Glucose and 40 IU/l human insulin. The second perfusion step was performed using the same collagenase P-containing Perfusion Solution II for digestion. Using one centrifugation step per liver tissue sample, two separate fractions of primary human hepatocytes were obtained.

PHH were isolated from liver tissue samples by a two-step EDTA/collagenase perfusion technique as described elsewhere (Kegel et al., 2016[17]) with the following modification: the liver tissue sample was cut into two approximately equal parts. Each part was cannulated and perfused in parallel using a pericyclic pump Cyclo 2 (Carl Roth, Karlsruhe, Germany) with two pump heads connected to Perfusion Solution I without (w/o G+I) or with (w G+I) 11 mM D-Glucose and 40 IU/l human insulin (Figure 1(Fig. 1)). The glucose and insulin concentration based on identical concentrations used in the PHH culture medium for later cell culture. The second perfusion step was performed using the same collagenase P containing Perfusion Solution II for digestion of both liver tissue pieces. In the ongoing isolation procedure, the liver tissue pieces were treated individually. The resulting PHH fractions were washed with PBS and resuspended in PHH culture medium. Afterwards, the cell number and viability was determined in a Neubauer counting chamber by Trypan blue. The cells were snap-frozen and stored at -80 °C for later characterization or seeded on cell culture plastics (Supplementary Figure 1).

### Tissue and cell lysis

PHHs were treated with lysis buffer consisting of PBS supplemented with 0.1 % SDS, 0.5 % Triton X-100 and 50 mM Trizma HCL and frozen at -20 °C. Cultured cells were lysed by freeze-thaw cycles and protein samples were collected by scratching using a cell scraper. In contrast, liver tissue samples were thawed and disrupted using a TissueLyser 2 (Qiagen, Hilden, Germany) at a speed of 30 Hz for 1 min. Samples were resuspended in ddH_2_O and sonicated for at least 10 min (Sonorex RK 100, Bandelin, Berlin, Germany). Aliquots were taken for protein and glycogen quantification.

### Biochemical glycogen assay

Glycogen content was determined quantitatively by an enzymatic cleavage in glucose following glucose determination closely related to the method of Lust et al. (1975[26]). Glycogen was measured in samples from supernatants from lysed cells or tissue. Glycogen content was normalized on protein content measured from the same sample using Bicinchoninic acid assay (BCA). A serial calibration curve using glycogen from oyster with a concentration range of 125 µg/ml to 6 mg/ml in ddH_2_O was prepared. Calibration standards, samples and blank were treated with 7 % perchloric acid in ddH_2_O (pH 1.2) and suspended carefully to terminate remaining enzyme functions. The reaction mixtures were alkalized by adding 0.5 M sodium hydroxide (Carl Roth, Karlsruhe, Germany) and heated in covered tubes to 100 °C for 10 min (Thermomixer pro, CellMedia, Elsteraue, Germany). The reaction tubes were centrifuged at 500 x g for 30 sec at 4 °C (Centrifuge 5424 R, Eppendorf) and 2 M acetate buffer (pH 4.5) containing 4 mg/ml amyloglucosidase was added to each cooled solution. All reaction mixtures were incubated at 55 °C for 2 h. Glucose determination was performed with Fluitest Glucose-Assay (Analyticon, Lichtenfels, Germany) according to the manufacturer's instructions.

### Determination of protein content

Protein content of frozen PHH cell pellets was determined using a BCA assay. A serial calibration curve using bovine serum albumin (BSA) in a concentration range of 31.25 µg/ml to 2 mg/ml was prepared. The samples were diluted to a concentration in the range of the calibration standard. Triplicates of sample, calibration standard or ddH_2_O (blank) were transferred to a 96-well plate (BD Falcon, Heidelberg, Germany or Greiner Bio-One, Frickenhausen, Germany). All wells were incubated with the reaction mixture consisting of BCA reagent A and 4 % copper sulfate solution in a ratio of 1:50, respectively. After incubating for 30 min in the dark, the absorbance was measured at a wavelength of 563 nm using a microplate reader (FLUOstar OPTIMA, BMG Labtech, Ortenberg, Germany or Synergy H1, BioTek, Bad Friedrichshall, Germany).

### Culture of hepatocytes

PHHs were cultured for up to 48 h in a classic 2D culture. Therefore, PHHs were seeded at a density of 110000/cm^2^ on rat tail collagen-coated cell culture plates (Greiner Bio-One, Frickenhausen, Germany) in PHH culture medium. Experiments were performed after an initial adherence period of 14-16 h (T_0_) and additional 24 h (T_1_) and 48 h (T_2_) (Supplementary Figure 1).

### Oil Red O staining

The diazo dye visualizes intracellular triacylglycerides - in PHH 16 h after seeding (T_0_), cell culture medium was removed and cells were washed twice with PBS. Cells were fixed with 4 % Roti^®^-Histofix (Carl Roth, Karlsruhe, Germany) for 30 min. Cells were gently washed two times with ddH_2_O and once for 5 min with 60 % 2-propanol (≥ 99.5 %, Carl Roth, Karlsruhe, Germany) in ddH_2_O. After allowing the cells to dry, they were incubated for 20 min at room temperature with filtered Oil Red O working solution. For this, an Oil Red O stock solution consisting of 3.5 mg/ml Oil Red O in 2-propanol (≥ 99.5 %) was mixed with ddH_2_O in a ratio of 3:2. After the incubation period, cells were washed three times with ddH_2_O to remove non-fixed dye. Finally, cells were dried and the Oil Red O stain was extracted with 2-propanol for 5 min with gentle rocking. Absorbance was measured at 550 nm using a microplate reader. For normalization, protein content was evaluated using sulforhodamine B (SRB) protein staining.

### Sulforhodamine B (SRB) protein staining

The protein dye SRB binds basic amino acids residues, so the results are linear with cellular protein and cell number. After the previous Oil Red O assay, cells were washed twice with ddH_2_O and covered with SRB solution for 30 min at room temperature with gentle rocking and protected from light. Unbound SRB was removed by washing four times with 1 % acetic acid (Carl Roth, Karlsruhe, Germany) in ddH_2_O. For quantification, SRB was extracted from cells under mild basic conditions with 10 mM un-buffered TRIS solution (pH 10 - 10.5) incubating 10 min at room temperature by gentle rocking. Samples were diluted in a ratio of 1:10 to 1:20 and 100 µl of these solutions were transferred in triplicates to a 96-well plate. TRIS solution was used as an assay control. Absorbance was measured at a wavelength of 565 nm due to SRB concentration and at 690 nm as reference wavelength using a microplate reader.

### XTT viability assay

The Cell Proliferation Kit II (Roche, Basel, Switzerland) is a colorimetric cell viability assay that detects cellular metabolic activity. During the assay, the yellow tetrazolium salt XTT (2.3-Bis-(2-Methoxy-4-Nitro-5-Sulfophenyl)-2H-Tetrazolium-5-Carboxanilide) is reduced to a highly colored formazan dye in the presence of an electron-coupling reagent by dehydrogenase enzymes in viable cells. Thus, the amount of the formazan produced is proportional to viable cells in the sample. For this, 16 h after seeding (T_0_), cell culture medium was removed, cells were washed twice with PBS and starved for 4 h in PHH culture medium without FCS. After that, PHH starvation medium and XTT labeling mixture were added to each well according to manufacturer's instructions and incubated for 6 h at 37 °C. Finally, 100 µl of these solutions were transferred in triplicates to a 96-well plate and the absorbance of the formazan dye was quantified at 450 nm using a microplate reader. For normalization, protein content was evaluated using SRB protein staining.

### Determination of urea

For urea determination in the supernatant of cultured PHHs, an assay according to Zawada et al. (2009[39]) was used. In brief: Both O-Phthalaldehyde and primaquine working solutions, containing 30 % Brij™-35 (Thermo Scientific, Schwerte, Germany) were prepared and diluted with ddH_2_O. A calibration curve of urea was prepared in a concentration range of 0.0035 mM to 4.16 mM by serial dilution. PHH culture medium was fully processed as the samples and used as an additional control. The assay was performed by placing 50 μl of sample, calibration standard, medium or ddH_2_O (blank) per well in triplicates in a 96-well cell culture plate (BD Falcon, Heidelberg, Germany). 100 μl of O-Phthalaldehyde reagent was added and the plate was shaken gently. Afterwards, 100 μl of primaquine reagent were transferred into each well and incubated for 1 h at 37 °C in the dark. The absorbance was measured at a wavelength of 430 nm using a microplate reader. Experiments were performed after an initial adherence period of 14-16 h (T_0_) and additional 24 h (T_1_) and 48 h (T_2_).

### Determination of albumin

Albumin, secreted to cell culture supernatants, was measured with an ELISA (Bethyl Laboratories, Montgomery, USA) according to the manufacturer's instructions. PHH culture medium was used as an assay control. Experiments were performed after an initial adherence period of 14-16 h (T_0_) and additional after 24 h (T_1_) and 48 h (T_2_).

### Determination of phase I and II enzyme activities

The measurement of enzyme activities of phase I and phase II enzymes were performed according to Collier et al. (2000[6]) and Donato et al. (2004[8]). Briefly, stock solutions of substrates and fluorescent metabolites were prepared in dimethyl sulfoxide (DMSO) and stored at -20 °C. PHHs were cultured as described above and medium was changed to starvation medium (without FBS and penicillin/streptomycin), 4 h prior the assay performance. Cells were treated with non-fluorescent substrates for phase I, which were converted to fluorescent metabolites by Cytochrome P450 enzymes (CYP), and fluorescent substrates for phase II, which undergo glucuronidation or sulfation (substrates are listed in Supplementary Table 2). Each incubation was performed in duplicates for 90 min. The medium supernatant was transferred to a new 96-well plate afterwards. For an absolute quantification, calibration curves were prepared with the metabolites of phase I/substrates of phase II and measured in the same plate as the samples. Wells with fixed hepatocytes were used as negative controls.

For the exclusive evaluation of phase I enzyme activity, the formed phase II metabolites were cleaved by an incubation with 100 U/ml β-glucuronidase and 100 U/ml sulfatase for 2 h. The increasing emissions of the fluorescent metabolites were determined at specific wavelengths using a microplate reader.

In contrast to CYP enzyme activities, the phase II enzyme activities were determined with fluorescent substrates which were converted to non-fluorescent metabolites. Additionally to the phase I products, 4-methylumbelliferone (4-MU) for estimating glucuronosyl-O-transferase activity and monochlorobimane (MCB) for glutathione-S-transferase (GST) activity were used. GST activity was relatively estimated since MCB shows no fluorescence and the fluorescent MCB conjugate was commercially not available.

Experiments were performed after an initial adherence period of 14-16 h (T_0_) and additional after 24 h (T_1_) and 48 h (T_2_).

### Statistical analysis

Data were analyzed by two-way ANOVA and unpaired t-test with Welch's correction using Graph Pad Prism 6 software. Results are given as mean ± SD. Differences were considered as significant at *p* < 0.05. Experiments were performed in PHHs or tissue from different donors (N) and data were measured in technical replicates (n) as stated in figure legends.

## Results

### Human liver tissue samples obtained from liver resections revealed low glycogen storage

**Figure 2:**
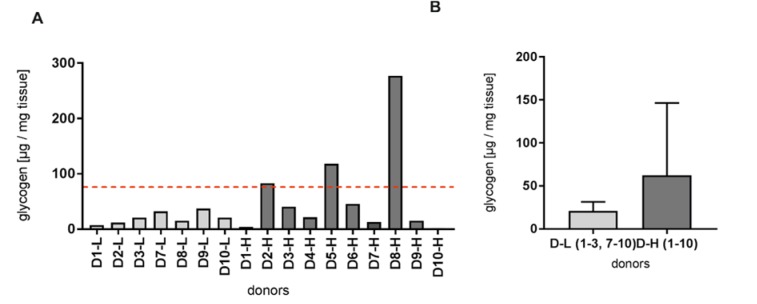
Glycogen content in human liver tissue. Amount of glycogen was measured in fresh tissue before the isolation procedure for single donors (A) and grouped for donors with low and high body mass index (B). The red dashed line indicates the average glycogen amount in the liver of approximately 8 % (A).

We hypothesized that the surgical procedure and postsurgery ischemia time during the isolation process lead to the emptying of glycogen storages. Therefore, we analyzed liver tissue samples after hemihepatectomy of patients with low and high BMI (Supplementary Table 1) for their glycogen content (Figure 2(Fig. 2)). Considering that full glycogen storage corresponds to approximately 8 % of the liver weight (Cole and Kramer, 2016[5]), the majority of analyzed human liver tissue samples revealed decreased levels of glycogen. Donors with a high BMI showed varying glycogen levels but, on average, higher glycogen values compared to samples from patients with low BMI. The glycogen storages of patients with low BMI are only filled up to approximately 10 % of the maximum storage capacity.

### Supplementation of glucose and insulin during hepatocyte isolation improves the viability of hepatocytes

We tested whether supplementation with glucose and insulin during the isolation process is capable of influencing the isolation outcome. Therefore, we perfused the liver tissue samples with perfusion solution I containing glucose and insulin for at least 20 min as part of the isolation process in comparison to the established buffer without glucose and insulin (Figure 1(Fig. 1)). This supplementation was only performed in perfusion step I; hence, the cells have time for the uptake of glucose and insulin, and an impact of metabolism can be observed.

Taken together, 12 donors with different BMIs were investigated in this study. All (N = 12) were analyzed for the isolation outcome. Because the yield of isolated PHHs limited their use for characterization, the study was split into two substudies. Substudy 1 (N = 6 D1-3-L & D1-3-H) investigates the effect of supplementation with glucose and insulin on PHH quality, while substudy 2 (N = 6 D4-6-L & D4-6-H) investigates the effect of the modified protocol on the regeneration of hepatic functions of PHHs in culture.

**Figure 3:**
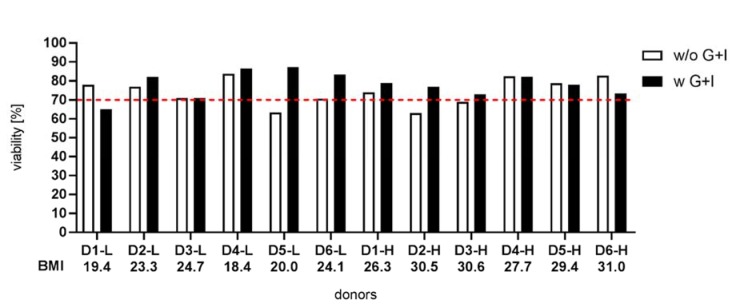
Effect of perfusion without and with glucose/insulin supplementation on viability of isolated primary human hepatocytes. Cell viability was determined via trypan blue exclusion technique. The red dashed line indicates 70 % viability as a quality criterion of hepatocytes (Godoy et al., 2013). For statistical analysis see Supplementary Figure 2.

The characterization of freshly isolated PHHs (N = 12) revealed predominantly higher viabilities of PHHs after supplementation with glucose and insulin (Figure 3(Fig. 3)). In detail, PHHs of 7 isolations showed an increase in viability, PHHs of 3 isolations exhibited no effect and PHHs from 2 isolations had a decrease in viability when isolated with the modified protocol. In 4 cases, supplementation with glucose and insulin helped low-viability cells to exceed 70 %, which is a generally accepted quality criterion for many PHH applications (Godoy et al., 2013[11]). Although the modified protocol revealed a clear trend to improve PHH viability, the statistical analysis did not show a significant result due to the varying values within a small range (Supplementary Figure 2). In contrast, an influence on the yield of isolated PHHs was not observable (Supplementary Figure 3).

### Cell quality of PHHs is influenced by their lipid content

**Figure 4:**
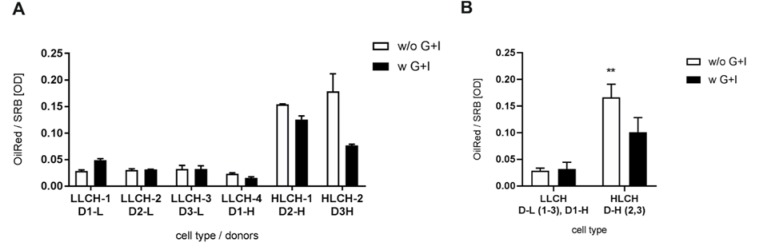
Impact of donors BMI on lipid content of hepatocytes after isolation procedure without and with glucose/insulin supplementation of perfusion solution I. Lipid contents were measured with Oil Red O staining. Data are normalized on protein content via sulforhodamine B protein staining. Data are presented as mean ± SD, plotted as technical replicates (n = 3) (A). Data were summarized by their lipid content and are presented as mean ± SD, plotted as low lipid containing hepatocytes-group (LLCH, N = 4, n = 3) and high lipid containing hepatocytes-group (HLCH, N = 2, n = 3) (B). Single groups were analyzed by unpaired T-Test with Welch's correction (** *p *= 0.0015).

Within the scope of substudy 1, PHHs of six donors, half with low (D1-3-L) and half with high BMI (D1-3-H) were seeded and investigated in detail. The lipid content of the adherent PHHs was characterized using Oil Red O staining normalized to protein content measured by the SRB assay. Quantification of the relative lipid content of isolated PHHs revealed that a low BMI correlated with low lipid content. In contrast, only two out of three donors with high BMI showed increased lipid content (Figure 4(Fig. 4)). The PHHs from donor D1-H showed notably low lipid levels despite a BMI of 26.3, and although the resected liver tissue was clearly classified with hepatic steatosis higher than 25 % by the pathological unit from University Medicine Leipzig (Supplementary Table 1). Therefore, the PHHs from donor D1-3-L, as well as D1-H, were regarded as low-BMI samples and classified as low lipid-containing hepatocytes (LLCH). PHHs from D2-3-H were grouped as high lipid-containing hepatocytes (HLCH). In LLCH, supplementation with glucose and insulin showed no or only a small change in the lipid content when isolated with the modified protocol. In contrast, the HLCH of the two donors showed a drop in the lipid content when supplemented with glucose and insulin during the cell isolation procedure.

### Supplementation of glucose and insulin during hepatocyte isolation improves the adherence of hepatocytes

**Figure 5:**
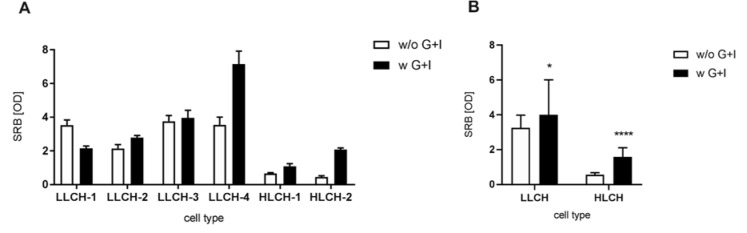
Effect of perfusion without and with glucose/insulin supplementation on adherence of isolated primary human hepatocytes (PHH). Cell adherence was measured with sulforhodamine B protein staining. Data are presented as mean ± SD, plotted as technical replicates (n = 9) (A). Data are presented as mean ± SD, plotted as low lipid containing hepatocytes-group (N = 4, n = 9) and high lipid containing hepatocytes-group-group (N = 2, n = 9) (B). Single groups were analyzed by unpaired T-Test with Welch's correction (*p = 0.0369, **** p < 0.0001).

Characterization of seeded PHHs revealed a positive effect on plating efficiency for the majority of PHHs isolated with a supplementation with glucose and insulin (Figure 5(Fig. 5)). There was a direct correlation between the adherence ability and the viability of hepatocytes. Again, PHHs isolated with supplementation of glucose/insulin from donors with high BMI showed a more pronounced improvement of the plating efficiency. However, only donor D1-L (LLCH-1) revealed a decrease in viability when supplemented with glucose and insulin and showed markedly decreased adherence.

### Hepatocytes with high lipid content profit from glucose/insulin supplementation showing a stabilization of energy metabolism

**Figure 6:**
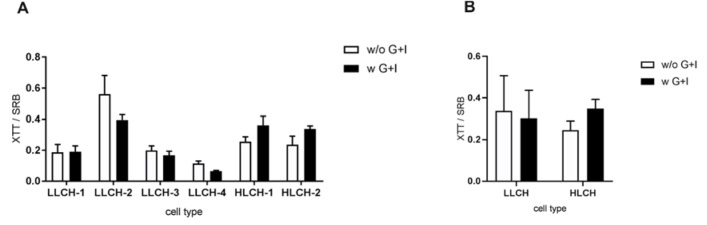
Effect of perfusion without and with glucose/insulin supplementation on primary human hepatocyte (PHH) activity in culture. The XTT assay was performed after initial adherence (T_0_) reflecting the cell activity differences directly after PHH isolation. Data are presented as mean ± SD, plotted as technical replicates (n = 9) and normalized on cell adherence (sulforhodamine B protein staining) (A). Data were summarized by their lipid content and are presented as mean ± SD, plotted as low lipid containing hepatocytes-group (N = 4, n = 9) and high lipid containing hepatocytes-group-group (N = 2, n = 9) (B).

The quantification of glycogen in isolated LLCH showed, in the majority of cases, no effect of glucose and insulin supplementation on the glycogen content (Supplementary Figure 4). Only in HLCH from donor D2-H, who already had a moderate glycogen level, was the supplementation procedure able to increase the glycogen content. Additionally, measurement of the cell activity using the XTT assay revealed that LLCH showed mostly lower cell activity when isolated with glucose and insulin supplementation compared to no supplementation. In contrast, HLCH showed increased cell activity when isolated with the modified protocol (Figure 6(Fig. 6)).

Taken together, supplementation with glucose and insulin during the cell isolation procedure showed no general improvement of glycogen storage. An increase in the glycogen content was only observed when the glycogen level was already high. However, in the majority of PHHs, an increase in viability was measured after supplementation independently of the BMI. This finding suggests that the majority of PHHs took up glucose, leading to a reduction in isolation stress and an improvement in viability. The beneficial effect of glucose and insulin supplementation persists after seeding, leading to an increased adherence of PHHs. However, PHHs with high lipid content (HLCH) showed a general loss in plating efficiency, suggesting a connection between lipid content and adherence capacity.

### Energetic stabilization of PHHs has no impact on hepatocyte function in vitro

**Figure 7:**
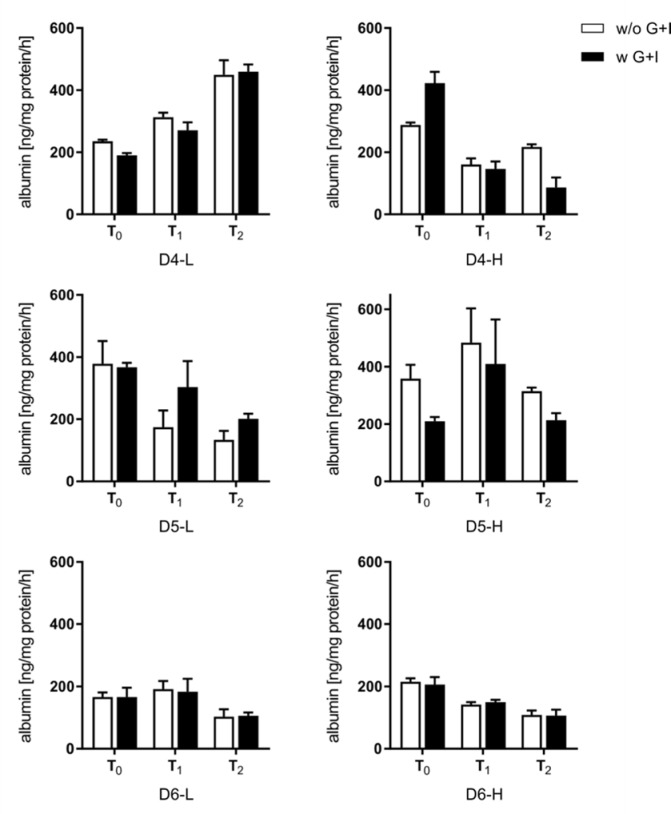
Synthesis rate of albumin of plated primary human hepatocytes (PHHs). Albumin secreted by PHHs was determined by ELISA after initial adherence (T_0_) and additional 24 h (T_1_) and 48 h (T_2_). Data are presented as mean ± SD, plotted as technical replicates (n = 3).

Within the scope of substudy 2, we investigated whether the improved viability and adherence after glucose/insulin supplementation also influenced the recovery of hepatocyte function in cell culture. Therefore, PHHs of six other donors with low (D4-6-L) and high BMI (D4-6-H) were cultured for 24 and 48 h, and the synthesis of albumin as a marker of protein synthesis and urea as a marker of metabolic capacity were measured. The initial albumin synthesis ranged between 200 and 400 ng/mg protein/h (Figure 7(Fig. 7)). The supplementation with glucose and insulin had no effect in the majority of PHH batches. However, two PHH batches from donors with high BMI showed different values. The initial (T_0_) albumin synthesis rate changed after 24 and 48 h and showed a similar course, independent of whether PHHs were isolated without or with glucose and insulin supplementation. Therefore, the changes in synthesis rates were donor-dependent and independent of BMI or supplementation with glucose and insulin.

**Figure 8:**
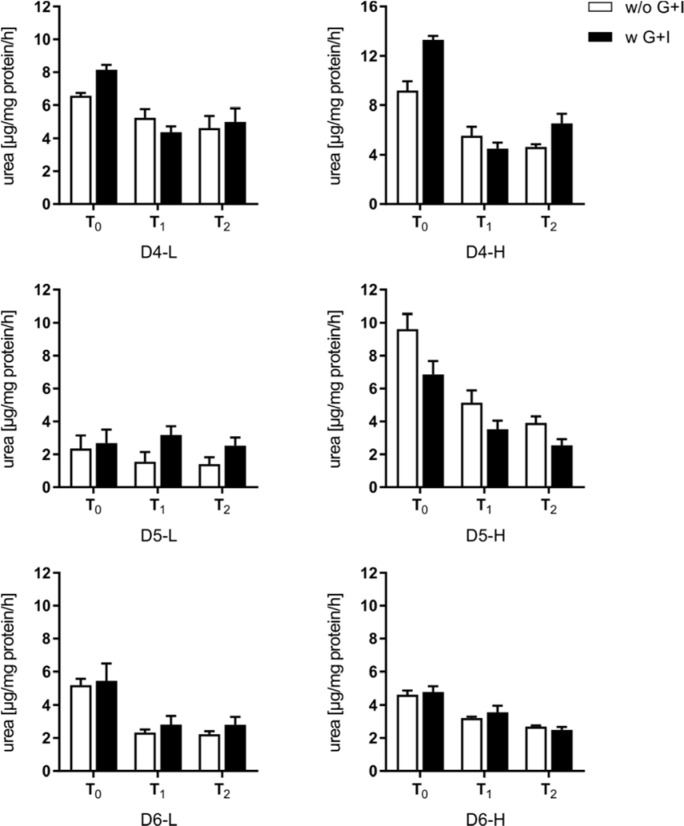
Synthesis rate of urea of plated primary human hepatocytes (PHHs). Urea secreted by PHH was determined after initial adherence (T_0_) and additional 24 h (T_1_) and 48 h (T_2_). Data are presented as mean ± SD, plotted as technical replicates (n = 3).

Similarly, the initial urea synthesis revealed donor-dependent capacities between 2 and 12 µg/mg protein/h (Figure 8(Fig. 8)). The glucose and insulin-supplemented PHHs showed similar or higher initial (T_0_) values of urea synthesis. Only in one donor (D5-H) the initial (T_0_) metabolic activities decreased to a lower value after glucose/insulin supplementation. PHHs from donors with low BMI were able to keep their initial urea synthesis stable for 48 h, while in PHHs from donors with high BMI, the urea synthesis further decreased in a time-dependent manner.

**Figure 9:**
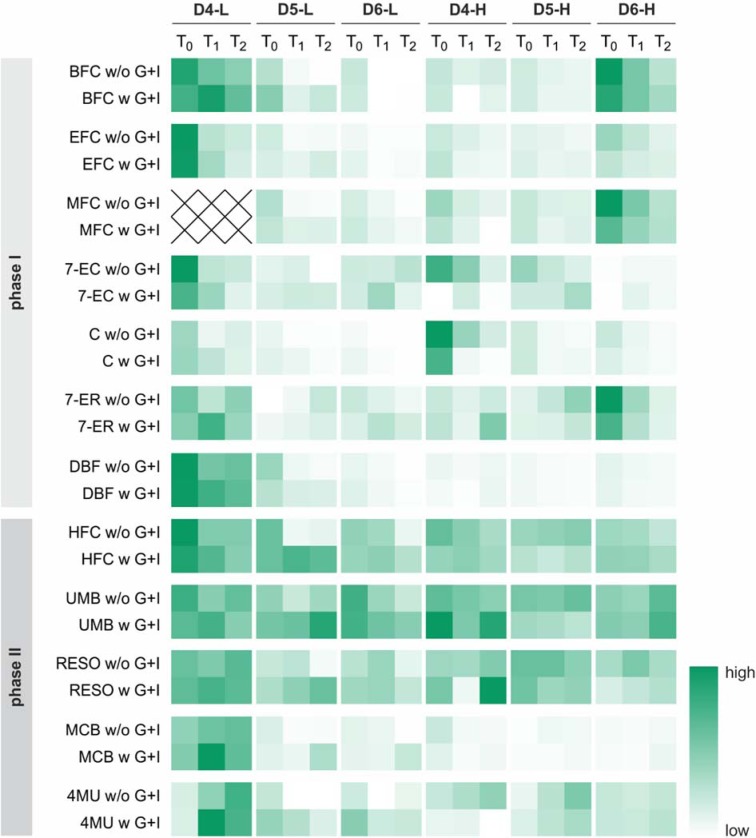
Determination of phase I and phase II enzyme activities of plated primary human hepatocytes (PHHs). Enzyme activity of PHHs was determined after initial adherence (T_0_) and additional 24 h (T_1_) and 48 h (T_2_). The substrates are listed in Supplementary Table 2 and the corresponding absolute values in Supplementary Tables 3 and 4. The activity is expressed as relative value from low activity (white) to high activity (dark green).

Enzymes of xenobiotic metabolism are markers of hepatic differentiation. Therefore, the activities of CYP450 enzymes as markers for phase I reactions, as well as selected conjugation reactions, as markers for phase II reactions were measured after an adherence phase (T_0_) and an additional 24 (T_1_) and 48 h (T_2_) using fluorescent probes (Figure 9(Fig. 9), Supplementary Tables 3 and 4). Two out of six donors (D4-L, D6-H) showed high CYP450 enzyme activities independently of BMI. Glucose and insulin-supplemented PHHs showed lower initial activities compared to conventionally isolated PHHs. In the majority of cases, CYP450 activities decreased time-dependently and independently of BMI or isolation strategy. In contrast, the initial phase II activities showed more equally distributed high values with smaller donor-dependent variations. One exception was the enzyme GSH transferase (substrate: MCB, Supplementary Table 4), which showed high activities in PHHs from one donor only. For the most part, the phase II activities were maintained or increased during the culture time. The supplementation with glucose and insulin showed no influence on the maintenance of the phase II activities.

Taken together, the investigation of hepatic function showed high donor variability in protein synthesis, metabolic activity and xenobiotic metabolism. While supplementation with glucose and insulin initially leads to higher urea synthesis capacities and lower CYP activities, no differences in the initial albumin synthesis rate and phase II reactions were observed. Cell cultivation for 48 h revealed that supplementation with glucose and insulin showed no late improvement of hepatic functions or maintenance of hepatic differentiation.

## Discussion

Despite the limited availability of primary human hepatocytes, they are still the gold standard for *in vitro* investigations of xenobiotic metabolism and hepatotoxicity. The improvements and novel developments in the field of liver surgery additionally affect the scarcity of tissue samples suitable for liver cell isolation (Horner et al., 2016[15]; Kegel et al., 2016[17]; Kluge et al., 2016[21]; Lloyd et al., 2004[25]). Therefore, further adaptation and optimization of liver cell isolation procedures are crucial. In the current work, we showed that viability and adherence could be improved by supplementing perfusion solution I with glucose and insulin.

### Influence of supplementation on initial hepatocyte quality

The quality of isolated liver cells depends on patient-related factors (Lee et al., 2014[24]) and intraoperative (Richert et al., 2004[32]) and postsurgery (Isaksson et al., 2011[16]) ischemia times. Intraoperative investigations of ischemia-reperfusion injury during portal clamping revealed anaerobic metabolism, increased glycogenolysis and cellular membrane damage (Isaksson et al., 2011[16]). Therefore, we investigated the availability of energy equivalents in liver tissue from resected livers. As the availability of energy equivalents depends on glycogen but also on lipid storage, we hypothesized a correlation with the systemic energetic state of the patient expressed by the BMI.

The characterization of human liver tissue samples of patients with low and high BMI revealed decreased levels of glycogen in almost all investigated samples, considering that full glycogen storage corresponds to 8 % of liver weight (Cole and Kramer, 2016[5]). Donors with a high BMI showed varying glycogen levels but, in the majority of cases, higher glycogen values compared to samples from patients with low BMI. The low BMI samples had approximately 10 % of the full glycogen capacity.

As hepatocytes require glucose and glycogen to survive warm and cold ischemia, as well as cellular stress during the isolation process, we hypothesized that supplementation with glucose and insulin during the isolation process could help to improve cell quality. The supplementation of the perfusate with glucose was performed before and is part of some published protocols (Lee et al., 2013[23]). However, the additional use of insulin to stimulate glucose uptake has not been previously described. To directly compare the influence of glucose/insulin supplementation, we modified the isolation setup, allowing the parallel isolation of two pieces of one tissue sample with varying perfusion solutions.

The resulting cell populations isolated without or with supplementation of glucose and insulin showed that the modified procedure was able to improve the viability of PHHs. The results indicate that in more than half of the cases, glucose uptake was able to restore energy resources and to prevent cellular death. In contrast, our data showed no effect of the modified protocol on the PHH yield isolated from the liver tissue pieces. Cutting a liver tissue piece in half for our modified isolation procedure results in slightly different tissue sizes with highly varying tissue and vessel architecture leading to different perfusion and digestion efficiencies. Therefore, the cell yields can vary independently of the protocol used and are not usable for further analysis.

The investigation of intracellular neutral lipids revealed that only two donors with high BMI (>30) showed correlating high hepatic lipid levels. In contrast, the PHHs from D1-H showed low intracellular lipid levels, despite a BMI of 26.3, and that the resected liver tissue was clearly classified with hepatic steatosis higher than 25 % by the pathological unit of University Medicine Leipzig. Microscopic investigations on a whole steatotic mouse liver revealed a heterogenic distribution of liver fat (Schwen et al., 2016[34]). Therefore, a liver tissue sample from a patient diagnosed with steatosis can have a low lipid content. Consequently, hepatocytes from donor D1-H were regarded as a low-BMI sample, and our groups were classified as LLCH and HLCH for further characterization.

The subsequent analysis of the glycogen content of PHHs revealed that only in one donor (HLCH-2) could an increase in the glycogen content be achieved. This donor had already full glycogen storage before the cell isolation procedure, as shown by the glycogen measurement in the corresponding tissue, suggesting that its PHHs had sufficient energy resources, enabling the storage of additional glucose in the form of glycogen. In contrast, the inability to store the offered glucose during perfusion suggests that most of the donor tissues had an emergent energy demand. Cellular regeneration and isolation stress are major processes requiring sufficient energy resources. Due to hypoxia and reoxygenation during surgery and cell isolation, reactive oxygen species (ROS) are produced. This response varies for PHHs originating from normal tissue, normal resected tissue and diseased tissue (Bhogal et al., 2010[1]). Proteomic analysis of PHHs exposed to hypoxia and reoxygenation confirmed that peroxisomal detoxification and oxidative stress protection are energy-demanding processes linked to enhanced glucose and glycogen metabolism (Strey et al., 2010[35]). Metabolic analysis confirmed a decrease in antioxidative metabolites, citric acid cycle intermediates and several energetic metabolites in freshly isolated PHHs (Cassim et al., 2017[4]). Consequently, supplementation with glucose and insulin led to a viability gain in most of the isolated PHH batches. These data are in accordance with Zeilinger and colleagues showing *in vitro* that glucose supplementation protects hepatocytes from cell membrane damage during hypoxia/reoxygenation (Zeilinger et al., 1997[40]). Correlating with viability, the relative adherence was also improved after supplementation with glucose and insulin, which was more pronounced in HLCH. The adherence requires a turnover of the plasma membrane to regenerate surface proteins damaged by the proteolytic cell isolation procedure. As the membrane regeneration of the cells is energy-dependent, a low glucose level limits the adherence in stressed hepatocytes. Analysis of attached and non-attached cryopreserved human hepatocytes showed an altered energy metabolism towards a stress-related glycolytic phenotype in proteomic analysis (Ölander et al., 2019[30]) and a downregulation of adhesion proteins in transcriptomic analysis (Terry et al., 2007[36]). Therefore, energetically stabilized and glucose-supplemented PHHs could have an advantage regarding their adherence capacity.

However, the adherence was generally very low in HLCH, independent of the supplementation. The investigation of the lipid content revealed that supplementation with glucose and insulin during the PHH isolation procedure can lead to different lipid contents in PHHs. As the *de novo* lipogenesis of triglycerides from glucose requires hours, if not days (Davidson et al., 2016[7]), we do not expect that the short-term supplementation with glucose and insulin during the isolation procedure can alter the lipid content of hepatocytes. However, supplementation can influence the survival of PHH subpopulations with varying lipid content, leading to alterations in the overall lipid content. In this regard, supplementation with glucose and insulin showed no or only small changes in the lipid content in LLCH. In contrast, HLCH showed a drop in the lipid content when supplemented with glucose and insulin during the cell isolation procedure, suggesting that PHHs with low lipid content are rescued due to supplementation.

Furthermore, a massive decrease in adherence was observed in HLCH independent of the supplementation. Green and coworkers observed that after plating hepatocytes, the remaining viable cells floating in the media had approximately threefold higher triacylglycerol content than the adherent cells (Green et al., 2017[12]), showing that hepatocytes that exceed a certain amount of lipid content were unable to adhere. We therefore hypothesize that the number of PHHs with high lipid content is increased without supplementation, which is linked to an enrichment of PHHs with reduced adherence. In contrast, supplementation with glucose and insulin leads to survival of low lipid-containing PHHs and, consequently, better adherence.

In general, the adherence and viability of a cell population correlates with its cell activity. However, only HLCH showed a correlation of the cell activity with increasing viability and adherence of PHHs. Therefore, other factors may influence cell activity. Our isolated PHH suspension represents a mixed population of periportal and pericentral hepatocytes with different metabolic activities. As demonstrated by Halpern and coworkers, periportal hepatocytes in mice show that the oxidative phosphorylation pathway is expressed higher in periportal hepatocytes, where the oxygen concentration is higher (Halpern et al., 2017[14]). Therefore, the cell activity measured in relation to NADPH and NADH concentration, as well as dehydrogenase activity, should be higher in periportal hepatocytes. The results indicate that performing the cell isolation without or with supplementation of glucose and insulin may have an effect on enriching PHH subpopulations differing in their cellular activities. We expected that an initial improvement of the energy resources also affects the regeneration of the cells in culture. Additionally, an effect of the isolation procedure on the selection of PHH populations should also be observable through functional parameters.

### Influence of supplementation on hepatic functions

Therefore, we investigated differentiated hepatic functions of cultured PHHs isolated with and without supplementation of glucose and insulin. We decided to perform the investigations in a classic 2D culture because this culture was stable for at least 3 days (Knobeloch et al., 2012[22]). The initial (T_0_) synthesis of albumin und urea in PHHs isolated with and without supplementation of glucose and insulin showed no difference between both procedures in most PHH batches. During the ongoing cell culture (T_1_ and T_2_), half of the PHH batches were able to maintain or restore albumin synthesis capacities, while in the others, the albumin synthesis dropped. These observations were independent of the BMI of the donors. In contrast, the urea synthesis capacity shows a clear tendency to lose activity during the cultivation time using our cell culture protocol. Two donors (D4-H, D5-H) showed a striking identical pattern of the initial values for albumin and urea, suggesting that both synthesis activities can be traced to the same hepatocyte population. The elucidation of lobular liver functions revealed that urea synthesis is performed mainly in the periportal zone, whereas albumin is synthesized by hepatocytes over the whole sinusoid but shows higher activities in the periportal zone, as well (Halpern et al., 2017[14]). Consequently, the higher initial urea synthesis for the donors D4-L and D4-H suggests that supplementation with glucose and insulin can enrich the cell suspension with periportal hepatocytes. However, the general reduction of urea synthesis in the majority of donors indicates that oxygen-dependent periportal PHHs have either a shorter survival rate or lose these activities due to adaption or dedifferentiation. Several studies have shown that the stabilization or improvement of hepatic functions, such as production of urea or albumin, is oxygen-dependent (Gilglioni et al., 2018[10]; Kidambi et al., 2009[18]), and PHH cultures maintain hepatic functions when cultured in a physiological oxygen concentration (Guo et al., 2017[13]).

The investigations on CYP450 activities revealed the highest activities for the donors D4-L and D6-H. Both donors showed lower initial values when isolated with supplementation of glucose and insulin. PHHs from the other donors showed a similarly reduced activity for the majority of CYP450 when isolated with the modified protocol. Therefore, supplementation with glucose and insulin decreases CYP activities either by dedifferentiation or influences the percentage of CYP450-rich PHH subpopulations. Most CYP450 activities are located in the pericentral region of the liver lobule (Oinonen and Lindros, 1998[29]), which particularly applies for the isoenzymes CYP1A2, CYP2A6, CYP2C19, CYP2E1 and CYP3A4 investigated in our study (McEnerney et al., 2017[27]). Therefore, the results indicate that supplementation with glucose and insulin leads to a dilution of a CYP450-rich pericentral hepatocyte population with additional periportal hepatocytes that are low in CYP450 activity. In contrast, the measured phase II enzyme activities were more stable or regenerated during the cultivation time, and the supplementation with glucose and insulin showed no striking initial difference.

Many genes of phase II enzymes, e.g., several isoforms of glutathione-S-transferases and sulfotransferases, are expressed in pericentral hepatocytes, but specific isoforms of these metabolic pathways also appear exclusively in periportal hepatocytes (Braeuning et al., 2006[3]). Most of the hepatic glucuronosyltransferases show an expression over the whole lobule with a higher expression in pericentral hepatocytes (Halpern et al., 2017[14]). Thus, phase II metabolism is more equally distributed in general. As our assays did not allow us to distinguish between the specific conjugation pathways, the detection of differences in the hepatocyte subpopulation ratio cannot be expected. This conclusion is in line with our observations on unchanged phase II activities between isolation without and with glucose and insulin supplementation.

The results correspond with observations that periportal hepatocytes are representative of high energy-consuming functions, such as albumin and urea synthesis and gluconeogenesis. Additionally, these cells are more sensitive to low oxygen levels, leading to adaption, dedifferentiation and loss of viability (Gilglioni et al., 2018[10]; Kidambi et al., 2009[18]; Kietzmann, 2017[19]). In contrast, pericentral hepatocytes are characteristic of glycogen synthesis from glucose, lipid storage and xenobiotic metabolism and are less sensitive to hypoxia.

## Summary, Limitations and Isolation Recommendation

Taken together, patient parameters, the surgical procedure and tissue handling can decrease hepatic energy resources and can lead to cell stress and cell death. Our modified protocol for the isolation of PHHs restores hepatic energy resources. Even if the hepatic glycogen level was unchanged, an indirect improvement of the availability of energy was shown by increased viability, plating efficiency and partial cellular activity. The plating efficiency showed a striking inverse correlation with increasing lipid content of the hepatocytes. The initial supplementation-dependent differences in lipid storage, albumin and urea synthesis, as well as xenobiotic metabolism, originate from changes in the isolated hepatocyte subpopulations. Consequently, performing PHH isolation without supplementation of glucose and insulin affects PHHs with low energy resources that die during the isolation procedure or the early cell culture. Supplementation with glucose and insulin during the liver cell isolation procedure improves the survival rates of hepatocytes with low energy resources and shifts the ratio of different PHH subpopulations to a mixed population that is more representative of the entire liver lobule. However, the changed composition of isolated PHHs had no significant impact on the maintenance of hepatic function during a 60 h cultivation time.

The study is limited because the functional characterization showed varying and highly donor-dependent results, which are typical when working with PHHs. Therefore, we supported our limited statistical analysis with the individual values for each batch, allowing us to confirm results on different readouts for a broad range of characterization parameters. In this study, we focused on factors that influence the energy resources, as they are affected by ischemia and hypoxia/reoxygenation and were identified as a major problem that is exacerbated by modern surgical methods. Despite these limitations, the study draws some conclusions that are true for the majority of investigated batches.

Consequently, we recommend supplementation with glucose and insulin because it improves the majority of PHH batches and leads to a cell suspension that better reflects the PHH populations of the entire liver lobule. When parameters in cell characterization must be determined absolutely (as in CYP assays), the most stable time frame is 36 h after seeding the cells; otherwise, an analysis relative to a control group is recommended (treated cells vs. control/untreated cells).

Further research is needed to characterize the influence of the isolation procedure on the selection and the final ratio of hepatocyte subpopulations. Additionally, further research on the improvement in liver cell culture is warranted to maintain hepatic functions in more vulnerable hepatocyte populations. Hence, the optimization of existing isolation and cultivation protocols is a prerequisite for guaranteeing high quantities of high-quality PHHs.

## Notes

Georg Damm and Gerda Schicht contributed equally as first authors.

## Author contributions statement

GS, AZ, MK, TW and VK collected the samples and patient information. GS, AZ and MK performed experiments. GS, AZ and CR analyzed data and designed figures. GD and DS designed the study. NF performed statistical analyses. GS, AZ, CR, GD and DS wrote or edited the manuscript.

## Declaration of conflict of interest statement

The authors declare no potential conflicts of interest with respect to the research, authorship and/or publication of this article.

## Funding statement

The study was supported by the Federal Ministry of Education and Research (BMBF, Germany) within the research network Virtual Liver Network (VLN) [grant number: BMBF 0315741] and LivSys Transfer [grant number: FKZ 031L0119D].

## Acknowledgement

We cordially thank Mandy Richter at SIKT Leipzig and Anja Schirmeier at Charité Berlin for excellent technical assistance and the team from Leipzig University hospital for the fruitful collaboration. Additionally, we thank Madlen Matz-Soja for her feedback to the discussion. The authors furthermore would like to thank Fritz Seidl, M.A. Interpreting and Translating, for proofreading and language-editing the manuscript.

## Supplementary Material

Supplementary material

## Figures and Tables

**Table 1:**
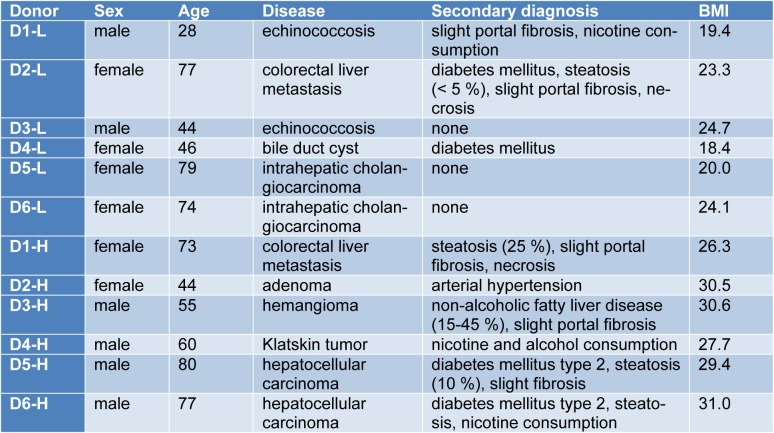
Donor data for tissue samples used for PHH isolation. Summary of donors including sex, age, medical history and body mass index (BMI). Samples were grouped according to BMI as low (L) with BMI < 25 or high (H) with BMI ≥ 25.
