# Supplementary material for: Effect of glucose and insulin supplementation on the isolation of primary human hepatocytes
Source: EXCLI J. 2019 Nov 18;18:1071–91. doi: 10.17179/excli2019-1782 (PMC6909377; doi:10.17179/excli2019-1782)
Supplement: Supplementary material [file EXCLI-18-1071-s-001.pdf]

EXCLI Journal 2019;18:1071-1091 – ISSN 1611-2156
Received: September 02, 2019, accepted: October 29, 2019, published: November 18, 2019

Supplementary material to:

EFFECT OF GLUCOSE AND INSULIN SUPPLEMENTATION ON THE ISOLATION OF PRIMARY HUMAN HEPATOCYTES

Georg Damm[1,2,3*], Gerda Schicht[1,2*], Andrea Zimmermann[1,2], Christiane Rennert[1,2], Nicolas Fischer[1,2], Melanie Kießig[3], Tristan Wagner[1], Victoria Kegel[1,3], Daniel Seehofer[1,3]

*These authors contributed equally.

1 Department of Hepatobiliary Surgery and Visceral Transplantation, University Hospital, Leipzig University, Leipzig, Germany
2 Saxonian Incubator for Clinical Translation (SIKT), Leipzig University, Leipzig, Germany
3 Department of General-, Visceral- and Transplantation Surgery, Charité University Medicine Berlin, 13353 Berlin, Germany

* Corresponding author: Dr. rer. nat. Georg Damm; Philipp-Rosenthal-Strasse 55, 04103 Leipzig, E-mail: georg.damm@medizin.uni-leipzig.de

http://dx.doi.org/10.17179/excli2019-1782

This is an Open Access article distributed under the terms of the Creative Commons Attribution License (http://creativecommons.org/licenses/by/4.0/).

EXCLI Journal 2019;18:XX-XX – ISSN 1611-2156
Received: September 02, 2019, accepted: October 29, 2019, published: XX, 2019

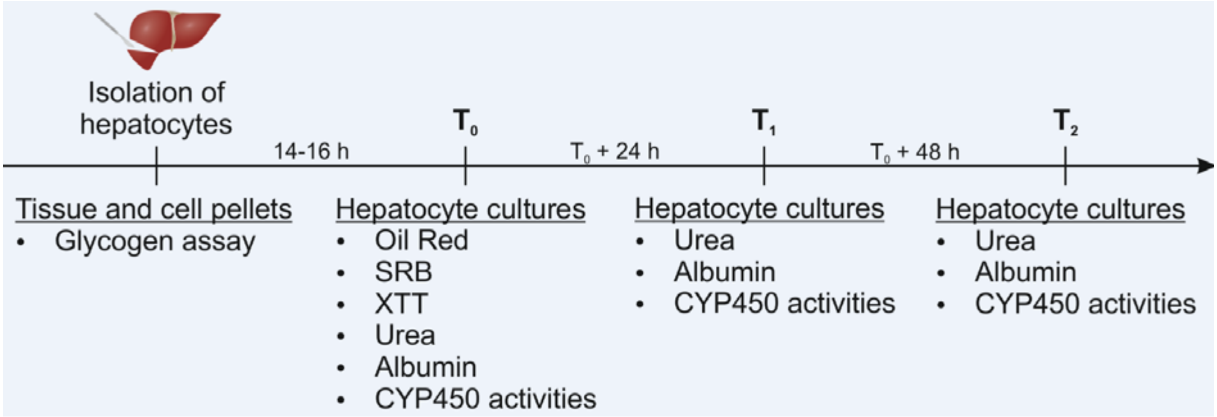

Supplementary Figure 1: Timeline of performed experiments. Tissue and freshly isolated PHH were used for glycogen assay. The cultured PHH were used for indicated assays after designated cultivation terms.

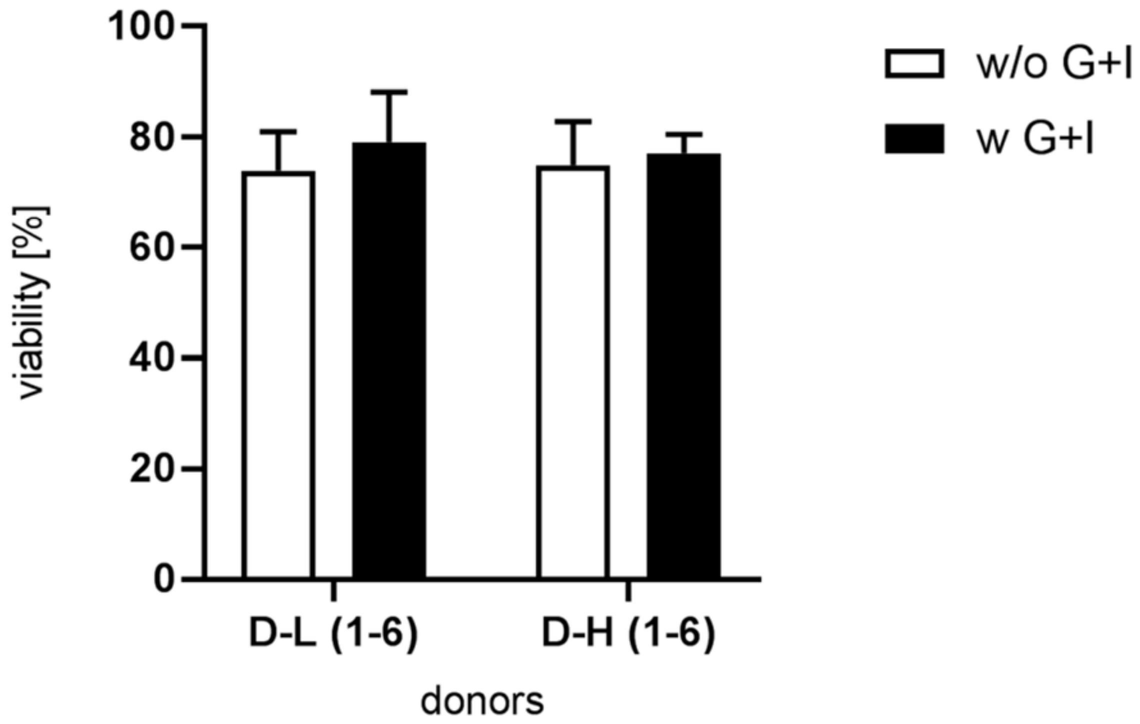

Supplementary Figure 2: Viability of freshly isolated hepatocytes. Supplementation of perfusion solution I with glucose and insulin seems to have no effect on viability of PHHs, directly measured after cell isolation. Data are plotted as mean ± standard deviation (N=6).

EXCLI Journal 2019;18:1071-1091 – ISSN 1611-2156
Received: September 02, 2019, accepted: October 29, 2019, published: November 18, 2019

A

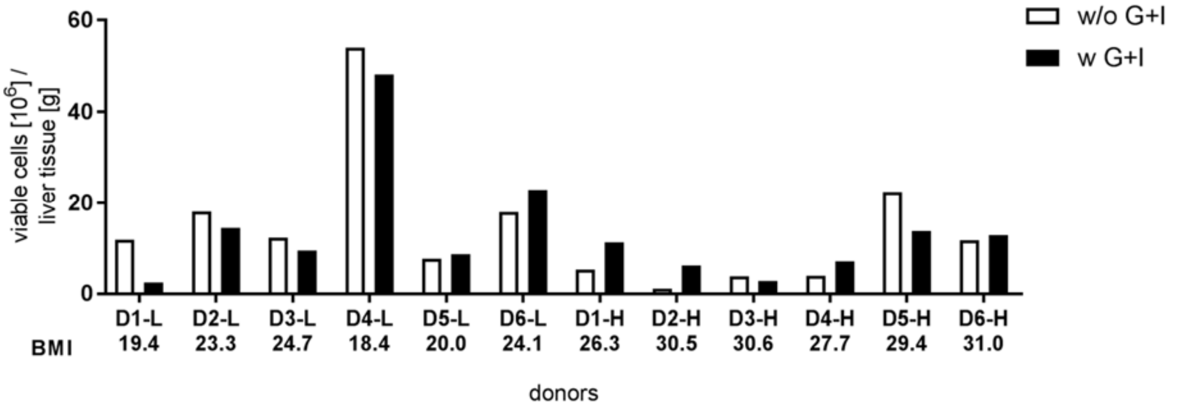

B

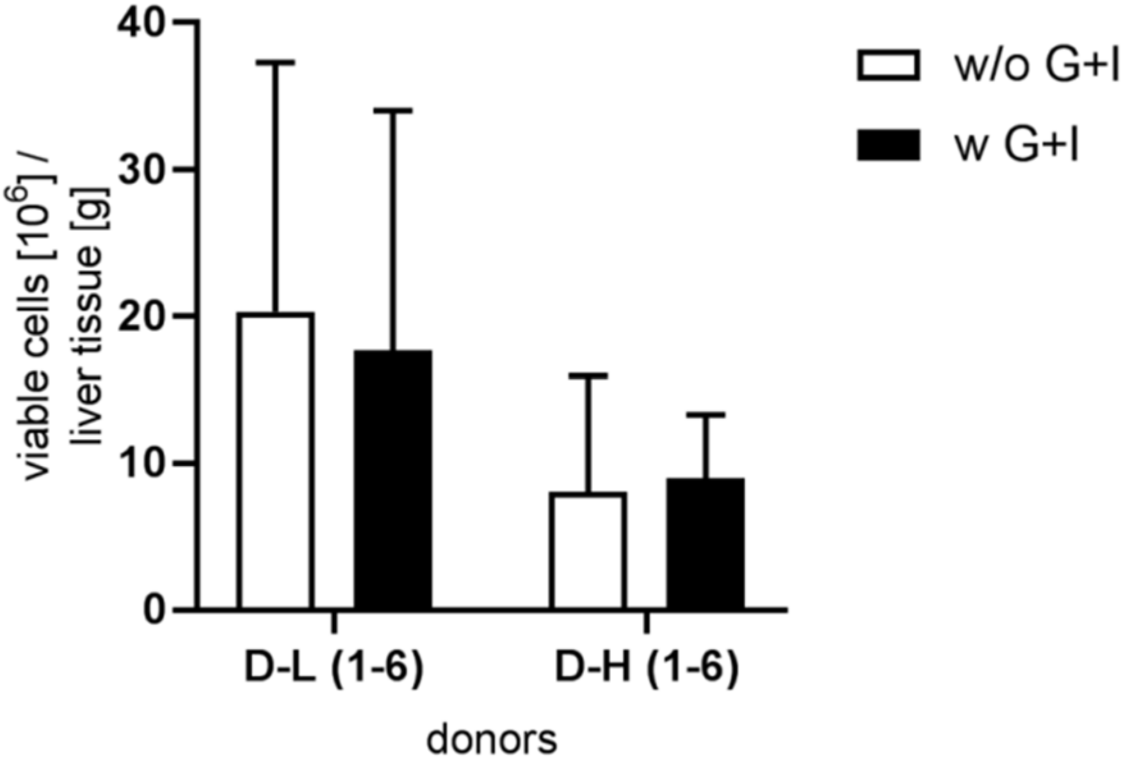

Supplementary Figure 3: Cell yield of freshly isolated hepatocytes. Supplementation of perfusion solution I with glucose and insulin seems to have no effect on cell yield of PHH, directly measured after cell isolation (**A**). There was no statistically significant interaction between BMI and supplementation of perfusion solution I (Two-way ANOVA, F (1, 20) = 0,1197, *p* = 0,7330). Data are presented as mean ± SD, plotted as replicates (N=6) (**B**).

EXCLI Journal 2019;18:1071-1091 – ISSN 1611-2156
Received: September 02, 2019, accepted: October 29, 2019, published: November 18, 2019

A

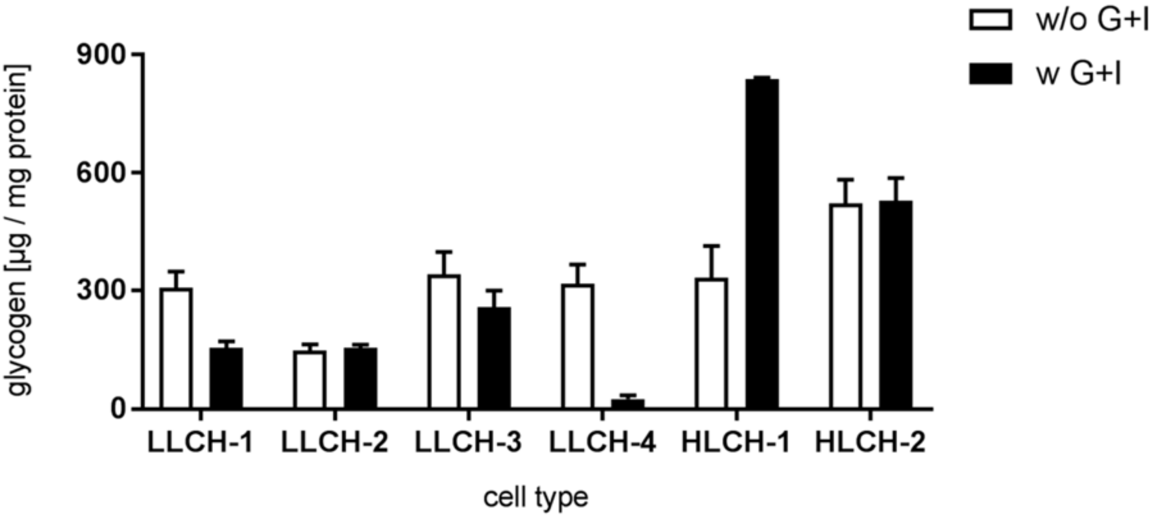

B

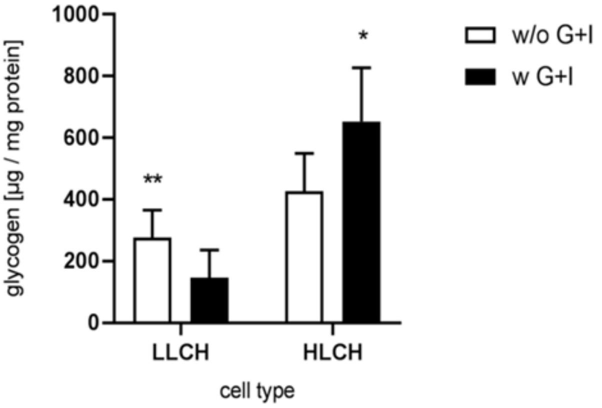

Supplementary Figure 4: Glycogen content of hepatocytes. Supplementation of perfusion solution I with glucose and insulin seems to have no effect on glycogen content of PHH. Glycogen content was directly measured after cell isolation. Data are normalized on protein content measured via BCA. There was a significant main effect for interaction (Two-way ANOVA, F (1, 31) = 19,96, p < 0,0001) and BMI (Two-way ANOVA, F (1, 31) = 67,68, p < 0,0001). Data are presented as mean ± SD, plotted as technical replicates (n = 3) (**A**). Data are presented as mean ± SD, plotted as LLCH-group (N = 4, n = 12) and HLCH-group (N = 2, n = 6) (**B**). Single groups were analyzed by unpaired T-Test with Welch's correction (* p = 0,0447, ** p = 0,0016).

Received: September 02, 2019, accepted: October 29, 2019, published: November 18, 2019

Supplementary Table 1: Summary of all donor data of liver tissues used for this study, including liver tissues used for glycogen analysis only and liver tissue used for PHH isolation. Donor data include sex, age, medical history and body mass index (BMI). Samples were grouped according to BMI as low (L) with BMI < 25 or high (H) with BMI ≥ 25.

Donor	Sex	Age	Disease	Secondary diagnosis	BMI
D1-L	male	28	echinococcosis	slight portal fibrosis, nicotine consumption	19.4
D2-L	female	77	colorectal liver metastasis	diabetes mellitus, steatosis (< 5 %), slight portal fibrosis, necrosis	23.3
D3-L	male	44	echinococcosis	none	24.7
D4-L	female	46	bile duct cyst	diabetes mellitus	18.4
D5-L	female	79	intrahepatic cholangiocarcinoma	none	20.0
D6-L	female	74	intrahepatic cholangiocarcinoma	none	24.1
D7-L	female	36	adenoma	none	23.0
D8-L	female	27	unclear liver tumor	slight portal fibrosis	23.8
D9-L	female	42	hemangioma	steatosis (25 %)	24.4
D10-L	male	49	liver metastasis	slight steatosis	24.8
D1-H	female	73	colorectal liver metastasis	steatosis (25 %), slight portal fibrosis, necrosis	26.3
D2-H	female	44	adenoma	arterial hypertension	30.5
D3-H	male	55	hemangioma	non-alcoholic fatty liver disease (15-45 %), slight portal fibrosis	30.6
D4-H	male	60	Klatskin tumor	nicotine and alcohol consumption	27.7
D5-H	male	80	hepatocellular carcinoma	diabetes mellitus type 2, steatosis (10 %), slight fibrosis	29.4
D6-H	male	77	hepatocellular carcinoma	diabetes mellitus type 2, steatosis, nicotine consumption	31.0
D7-H	male	72	liver metastasis	steatosis (40 %), slight fibrosis	25.0
D8-H	female	38	Klatskin tumor	biliary fibrosis	26.0
D9-H	male	76	hepatocellular carcinoma	steatosis	29.0
D10-H	male	54	colorectal liver metastasis	steatosis (40 %)	29.5

Supplementary Table 2: List of substrates, concentrations, detected fluorescent metabolites and catalyzing enzymes for (**a**) phase I assays and (**b**) phase II assays.

(**a**) substrates phase I assays

Substrate		Concentration	Fluorescent metabolite		CYP enzyme
BFC	7-Butyloxy-4-(trifluoromethyl)coumarin	100 µM	HFC	7-Hydroxy-4-(trifluoromethyl)coumarin	1A2, 3A4, 2B6, 2C19, 2C9
EFC	7-Ethoxy-4-(trifluoromethyl)coumarin	40 µM	HFC	7-Hydroxy-4-(trifluoromethyl)coumarin	2B6, 2E1, 2C19
MFC	7-Methoxy-4-(trifluoromethyl)coumarin	40 µM	HFC	7-Hydroxy-4-(trifluoromethyl)coumarin	2E1, 2C9
7-EC	7-Ethoxycoumarin	50 µM	UMB	Umbelliferone	1A2, 2E1
C	Coumarin	100 µM	UMB	Umbelliferone	2A6
7-ER	7-Ethoxyresorufin	12.5 µM	RESO	Resorufin	1A2, 1A1
DBF	Dibenzylfluorescein	20 µM	FLUO	Fluorescein	2C9, 2C8

(**b**) substrates phase II assays

	Substrate	Concentration
HFC	7-Hydroxy-4-(trifluoromethyl)coumarin	25 µM
UMB	Umbelliferone	20 µM
RESO	Resorufin	10 µM
MCB	Monochlorobimane	80 µM
4MU	4-Methylumbelliferone	100 µM

EXCLI Journal 2019;18:1071-1091 – ISSN 1611-2156
Received: September 02, 2019, accepted: October 29, 2019, published: November 18, 2019

Supplementary Table 3: Determination of (**a**) phase I and (**b**) phase II enzyme activity of donors with low (L) BMI. Enzyme activity of PHH was determined after initial adherence (T_0) and additional 24 h (T_1) and 48 h (T_2). Data are presented as means ± average absolute deviation in nMol/mg protein/h.

(**a**) phase I enzyme activity

**Substrate**	**Time**	**D4-L**		**D5-L**		**D6-L**	
		**w/o G+I**	**w G+I**	**w/o G+I**	**w G+I**	**w/o G+I**	**w G+I**
BFC	T_0	4.868 ± 0.416	4.137 ± 0.388	1.608 ± 0.078	2.639 ± 0.078	1.229 ± 0.055	1.089 ± 0.035
	T_1	3.211 ± 0.467	5.128 ± 1.048	0.233 ± 0.004	0.746 ± 0.004	0 ± 0	0 ± 0
	T_2	2.566 ± 0.092	3.460 ± 0.698	0.008 ± 0.008	1.262 ± 0.311	0 ± 0	0.015 ± 0.015
EFC	T_0	6.219 ± 0.960	6.052 ± 0.180	1.216 ± 0.138	1.019 ± 0.015	0.402 ± 0.324	0.703 ± 0.008
	T_1	1.743 ± 0.067	2.345 ± 0.168	0.208 ± 0.007	0.629 ± 0.045	0.093 ± 0.011	0.105 ± 0.057
	T_2	1.247 ± 0.067	1.053 ± 0.090	0.291 ± 0.137	1.123 ± 0.161	0.084 ± 0.020	0.220 ± 0.003
MFC	T_0			1.843 ± 0.143	1.486 ± 0.052	1.017 ± 0.169	1.180 ± 0.078
	T_1			0.281 ± 0.090	0.804 ± 0.070	0.439 ± 0.005	0.590 ± 0.003
	T_2			0.156 ± 0.128	0.813 ± 0.001	0.099 ± 0.006	0.337 ± 0.014
7-EC	T_0	1.739 ± 0.080	1.279 ± 0.101	0.195 ± 0.097	0.288 ± 0.144	0.346 ± 0.009	0.330 ± 0.165
	T_1	0.454 ± 0.200	0.702 ± 0.193	0.260 ± 0.000	0.352 ± 0.003	0.332 ± 0.166	0.682 ± 0.341
	T_2	0.382 ± 0.086	0.218 ± 0.190	0 ± 0	0.339 ± 0.041	0.485 ± 0.078	0.199 ± 0.038
C	T_0	5.789 ± 0.605	5.996 ± 0.465	1.332 ± 0.126	1.790 ± 0.182	0.597 ± 0.035	1.185 ± 0.170
	T_1	1.192 ± 0.307	3.777 ± 0.418	0.270 ± 0.046	1.180 ± 0.325	0.118 ± 0.001	0.315 ± 0.046
	T_2	2.246 ± 0.342	1.996 ± 0.091	0.129 ± 0.010	0.380 ± 0.074	0.023 ± 0.023	0.031 ± 0.018
7-ER	T_0	0.515 ± 0.009	0.438 ± 0.012	0 ± 0	0.061 ± 0.000	0.202 ± 0.003	0.137 ± 0.005
	T_1	0.235 ± 0.074	0.695 ± 0.187	0.053 ± 0.005	0.090 ± 0.006	0.119 ± 0.035	0.260 ± 0.004
	T_2	0.418 ± 0.027	0.365 ± 0.019	0.209 ± 0.007	0.136 ± 0.014	0.058 ± 0.000	0.163 ± 0.008
DBF	T_0	8.193 ± 0.504	8.074 ± 0.747	3.378 ± 1.082	2.342 ± 0.198	0.784 ± 0.008	1.086 ± 0.102
	T_1	4.617 ± 1.006	6.378 ± 0.293	0.577 ± 0.048	1.309 ± 0.140	0.381 ± 0.027	0.490 ± 0.062
	T_2	5.017 ± 0.609	5.366 ± 0.642	0.270 ± 0.090	1.213 ± 0.357	0.021 ± 0.001	0.141 ± 0.035

(**b**) phase II enzyme activity

Substrate	Time	D4-L		D5-L		D6-L	
		w/o G+I	w G+I	w/o G+I	w G+I	w/o G+I	w G+I
HFC	T_0	56.019 ± 0.930	51.052 ± 5.632	33.932 ± 4.962	34.169 ± 5.721	24.821 ± 1.575	23.549 ± 2.303
	T_1	28.917 ± 1.116	39.305 ± 1.651	3.88 ± 0.018	41.121 ± 11.119	21.387 ± 0.059	25.983 ± 1.745
	T_2	28.582 ± 0.928	27.152 ± 1.363	5.617 ± 0.406	36.984 ± 7.660	4.736 ± 0.313	16.757 ± 0.480
UMB	T_0	32.339 ± 2.077	27.527 ± 1.449	18.291 ± 3.775	23.103 ± 4.731	31.751 ± 2.024	31.194 ± 1.737
	T_1	19.708 ± 3.324	30.979 ± 4.129	9.063 ± 3.114	24.658 ± 7.260	16.608 ± 1.288	23.877 ± 2.863
	T_2	26.022 ± 1.073	20.051 ± 0.712	15.818 ± 0.953	35.967 ± 0.313	8.952 ± 1.499	19.736 ± 0.582
RESO	T_0	6.314 ± 0.148	6.767 ± 0.285	2.396 ± 0.699	3.387 ± 0.060	2.945 ± 0.331	4.010 ± 0.247
	T_1	5.362 ± 0.738	7.745 ± 0.392	2.847 ± 0.268	4.718 ± 0.482	4.257 ± 0.629	4.312 ± 0.351
	T_2	7.038 ± 0.873	6.928 ± 0.264	0.469 ± 1.211	6.344 ± 0.456	1.115 ± 0.329	2.521 ± 0.007
MCB	T_0	23.241 ± 3.928	26.325 ± 1.319	7.668 ± 0.917	6.325 ± 0.072	6.025 ± 0.007	5.619 ± 0.731
	T_1	30.458 ± 4.285	51.857 ± 3.130	1.127 ± 0.252	4.219 ± 0.039	4.110 ± 0.510	4.79 ± 0.212
	T_2	32.759 ± 1.547	33.44 ± 3.129	1.566 ± 0.618	17.376 ± 0.093	0.001 ± 0.001	12.092 ± 0.056
4MU	T_0	37.382 ± 7.091	35.698 ± 1.516	55.590 ± 11.842	97.411 ± 6.606	47.041 ± 0.406	111.313 ± 12.220
	T_1	104.69 ± 8.264	230.128 ± 24.386	0 ± 0	69.985 ± 15.523	0 ± 0	46.983 ± 27.485
	T_2	177.751 ± 8.354	170.956 ± 7.366	0 ± 0	33.845 ± 14.911	22.44 ± 8.914	50.491 ± 5.281

EXCLI Journal 2019;18:1071-1091 – ISSN 1611-2156
Received: September 02, 2019, accepted: October 29, 2019, published: November 18, 2019

Supplementary Table 4: Determination of (**a**) phase I and (**b**) phase II enzyme activity of donors with high (H) BMI. Enzyme activity of PHH was determined after initial adherence (T_0) and additional 24 h (T_1) and 48 h (T_2). Data are presented as means ± average absolute deviation in nMol/mg protein/h.

(**a**) phase I enzyme activity

**Substrate**	**Time**	**D4-H**		**D5-H**		**D6-H**	
		**w/o G+I**	**w G+I**	**w/o G+I**	**w G+I**	**w/o G+I**	**w G+I**
BFC	T_0	1.280 ± 0.090	1.179 ± 0.144	1.044 ± 0.040	1.030 ± 0.042	5.408 ± 0.563	4.753 ± 0.688
	T_1	0.733 ± 0.018	0 ± 0	0.611 ± 0.007	0.505 ± 0.010	2.974 ± 0.089	3.004 ± 0.000
	T_2	0.958 ± 0.002	0.625 ± 0.218	0.460 ± 0.051	0.483 ± 0.079	1.510 ± 0.180	1.984 ± 0.105
EFC	T_0	1.327 ± 0.416	1.627 ± 0.207	0.778 ± 0.039	0.924 ± 0.009	2.537 ± 0.009	1.612 ± 0.074
	T_1	0.876 ± 0.006	0.519 ± 0.013	0.629 ± 0.004	0.557 ± 0.096	1.491 ± 0.017	1.043 ± 0.034
	T_2	0.526 ± 0.059	0.420 ± 0.146	0.391 ± 0.037	0.635 ± 0.019	0.766 ± 0.040	0.878 ± 0.016
MFC	T_0	2.338 ± 0.110	1.655 ± 0.060	1.366 ± 0.006	1.371 ± 0.004	5.875 ± 0.403	4.063 ± 0.242
	T_1	1.032 ± 0.218	0.693 ± 0.160	0.863 ± 0.033	0.598 ± 0.066	3.162 ± 0.158	2.500 ± 0.048
	T_2	0.638 ± 0.001	0 ± 0	0.806 ± 0.013	0.826 ± 0.040	1.710 ± 0.087	1.837 ± 0.109
7-EC	T_0	1.363 ± 0.028	0 ± 0	0.741 ± 0.076	0.342 ± 0.075	0.022 ± 0.022	0.004 ± 0.004
	T_1	0.828 ± 0.032	0.337 ± 0.337	0.406 ± 0.123	0.344 ± 0.027	0.098 ± 0.036	0.192 ± 0.012
	T_2	0.256 ± 0.128	0.015 ± 0.546	0.249 ± 0.249	0.618 ± 0.062	0.087 ± 0.006	0.095 ± 0.010
C	T_0	14.738 ± 0.634	10.965 ± 0.904	2.935 ± 0.051	3.049 ± 0.027	3.244 ± 0.102	2.105 ± 0.025
	T_1	6.223 ± 0.587	0.868 ± 0.057	0.844 ± 0.031	0.860 ± 0.017	1.271 ± 0.079	1.036 ± 0.033
	T_2	2.610 ± 0.210	0.244 ± 0.065	0.566 ± 0.066	0.994 ± 0.069	0.519 ± 0.022	0.557 ± 0.179
7-ER	T_0	0.201 ± 0.015	0.248 ± 0.034	0.111 ± 0.006	0.122 ± 0.001	0.899 ± 0.021	0.675 ± 0.012
	T_1	0.110 ± 0.009	0.090 ± 0.010	0.212 ± 0.017	0.125 ± 0.006	0.350 ± 0.011	0.268 ± 0.008
	T_2	0.183 ± 0.013	0.466 ± 0.020	0.402 ± 0.003	0.213 ± 0.022	0.123 ± 0.006	0.113 ± 0.013
DBF	T_0	0.658 ± 0.132	0.334 ± 0.105	0.515 ± 0.012	0.498 ± 0.025	0.913 ± 0.090	0.805 ± 0.066
	T_1	0.327 ± 0.035	0.097 ± 0.025	0.298 ± 0.019	0.275 ± 0.004	0.548 ± 0.029	0.429 ± 0.045
	T_2	0.610 ± 0.032	0.720 ± 0.014	0.177 ± 0.006	0.161 ± 0.000	0.351 ± 0.007	0.354 ± 0.003

EXCLI Journal 2019;18:1071-1091 – ISSN 1611-2156
Received: September 02, 2019, accepted: October 29, 2019, published: November 18, 2019

(**b**) phase II enzyme activity

Substrate	Time	D4-H		D5-H		D6-H	
		w/o G+I	w G+I	w/o G+I	w G+I	w/o G+I	w G+I
HFC	T_0	35.212 ± 0.524	23.768 ± 0.309	22.628 ± 0.014	15.986 ± 0.450	22.210 ± 0.593	24.839 ± 1.044
	T_1	27.029 ± 0.674	26.422 ± 3.414	25.221 ± 1.104	12.527 ± 0.132	20.269 ± 0.414	24.214 ± 1.339
	T_2	19.534 ± 0.359	21.247 ± 2.049	27.557 ± 0.960	16.803 ± 1.557	13.959 ± 0.143	19.787 ± 0.021
UMB	T_0	26.612 ± 2.020	40.934 ± 5.624	22.717 ± 0.241	15.584 ± 0.155	19.059 ± 0.378	20.496 ± 0.952
	T_1	22.641 ± 0.162	21.744 ± 0.726	21.766 ± 0.070	14.303 ± 0.020	16.883 ± 0.839	19.079 ± 0.496
	T_2	19.427 ± 0.304	36.163 ± 4.999	25.437 ± 0.776	11.201 ± 0.171	27.202 ± 0.759	30.262 ± 0.084
RESO	T_0	4.082 ± 0.677	5.741 ± 1.433	6.348 ± 0.334	5.996 ± 0.549	3.652 ± 0.446	1.685 ± 0.046
	T_1	3.901 ± 0.129	0.718 ± 0.274	6.345 ± 0.102	4.122 ± 0.274	5.545 ± 0.135	2.449 ± 0.251
	T_2	5.179 ± 0.019	10.358 ± 1.746	4.858 ± 0.031	4.631 ± 0.507	3.632 ± 0.113	3.179 ± 0.139
MCB	T_0	11.229 ± 0.560	6.340 ± 1.529	0.780 ± 0.157	1.016 ± 0.105	2.144 ± 0.168	1.747 ± 0.127
	T_1	2.828 ± 0.252	1.779 ± 0.205	3.541 ± 0.287	1.353 ± 0.177	1.554 ± 0.358	1.016 ± 0.047
	T_2	2.080 ± 0.137	3.109 ± 0.451	2.789 ± 0.491	2.532 ± 0.131	2.836 ± 0.026	4.076 ± 0.350
4MU	T_0	52.787 ± 5.512	21.920 ± 21.92	21.488 ± 3.707	33.705 ± 0.180	53.682 ± 6.488	52.762 ± 1.375
	T_1	74.535 ± 14.348	23.250 ± 41.224	65.632 ± 7.488	61.335 ± 1.150	44.759 ± 2.329	49.265 ± 6.165
	T_2	103.601 ± 0.753	0 ± 0	121.177 ± 2.058	81.467 ± 11.288	55.456 ± 4.064	65.258 ± 3.422
